# The evolution of the emergency mental health system in Israel - from the 1980’s until today

**DOI:** 10.1186/s13584-015-0017-8

**Published:** 2015-07-15

**Authors:** Moran Bodas, Bella Ben-Gershon, Zohar Rubinstein, Tal Bergman-Levy, Kobi Peleg

**Affiliations:** The Department of Disaster Medicine, School of Public Health, Sackler Faculty of Medicine, Tel-Aviv University, Tel-Aviv, Israel; Mental Health Services Division, Ministry of Health, Jerusalem, Israel; The Mental Health Array, Home Front Command, Israel Defense Forces, Ramla, Israel; Israel National Center for Trauma & Emergency Medicine Research, The Gertner Institute for Epidemiology and Health Policy Research, Sheba Medical Center, Tel Hashomer, Ramat-Gan, Israel

**Keywords:** Mental Health, Psycho-trauma, Resilience, Emergency, War, Israel

## Abstract

Emergency and disaster situations such as war or terrorism can leave a devastating impact on the mental well-being of victimized populations. In Israel, the civilian aspects of trauma-related mental distress were first extensively tackled during the 1980s, and mainly within the terror-stricken Jerusalem and the localities along the northern border. Since then, a systematic process of trial and error has led to the evolution of emergency mental health services in the country. Over the course of about forty years, it has grown to be an exemplary one. It is a system deeply rooted in the ground, resulting from both a change of discourse and a naturalistic process of lesson learning, that is, drawing conclusions from actual fieldwork. This process and its implications on the mental well-being of Israelis are thoroughly discussed in this research.

## Background

Emergency and disaster situations, such as war or terrorism, can result not only in loss of life or damage to property and infrastructure, but also with a devastating impact on the mental well-being of the victimized populations. Exposure to life-threatening situations, either first or second handed, can increase anxiety and in some cases reach the status of Acute Stress Reaction (ASR). Untreated, these cases can deteriorate to Acute Stress Disorder (ASD) and even Post-Traumatic Stress Disorder (PTSD) [1,2].

In Israel, like in other places around the world, the issue of mental health in crisis was first studied within a military context. This includes ’Combat Reaction’ (also known in the October War as ‘shell shock’), mental reactions of Prisoner-of-War (POWs), and other battle trauma syndromes. In the early stages of mental care in Israel, the eligibility for state-subsidized psychotherapy was reserved for individuals who met certain criteria outlined in the national insurance law, which originally favored shell-shocked veterans and almost disregarded distressed civilians [3].

Civilian aspects of emergency mental health in Israel were first tackled in light of the frequent attacks by Hezbollah in Lebanon on the Northern-border settlements in the 1980s, particularly in the city of Kiryat-Shemona (See Appendix for additional information on this and other armed conflicts described in this paper). During those days, mental health services were provided to the public through the outreach of professional personnel to the different districts of the city. The outreach included shelter visitations, support groups and ‘get-togethers’ that were aimed at identifying the more severe mental cases, and referring them for specific treatment plans. Back then, most of this relief work was performed by voluntary associations and Non-Governmental Organizations (NGOs) such as The Community Stress Prevention Centre (a.k.a. “Mashabim”), with little to no governmental regulation.

An additional example of the efforts made to support the mental well-being of the victimized population were the intervention teams that were established following the April 22^nd^, 1979 terrorist attack on the northern city of Naharya. These intervention teams comprised of a combination of medical personnel and social workers, and financed by the Ministry of Welfare, could be dispatched on-call to any location in the northern border to assist with mental first aid. The concept of these teams was later adopted by local authorities, and was successfully implemented mostly in terror-stricken Jerusalem and the northern border settlements during the 1980s and 1990s [4].

The First Gulf War (1991) was one of the main catalysts to the introduction of the civilian mental resilience to the spotlight. [4] For the Israeli home front, this war involved nocturnal missile attacks on civilian targets, the absence of retaliatory strikes by the Israeli Defense Forces, the threat of a chemical attack, and the compulsory confinement of family members in air-sealed, sheltered areas during missile attacks [5]. While the society as a whole coped well with the unfamiliar threats, some portions of the population was in fact mentally affected by the war. Approximately 43% of the 773 casualties evacuated to hospitals were diagnosed as psychological casualties and an additional 27% had mistakenly injected themselves with atropine, which was provided for the civilian population as an auto-injected antidote in case of a chemical gas attack [6]. Some of the effects of the attack on the mental well-being of the Israeli people had far reaching consequences even long after the war was over [7].

### The establishment and evolution of the system

The Emergency Mental Health Services have their roots in the ambulatory services of the mental health clinics, psychiatric wards of general hospitals and the psychiatric hospitals in Israel. When the First *Intifada* (1987–1991) broke out and an increasing number of victims were in need of mental assistance, this service was provided through those existing channels. Overall, and mainly due to the maladaptation of this system to victims of mass-trauma, the service provided for psycho-traumatized patients was relatively poor. In addition, substantial stigmatization of patients was involved. It became increasingly evident that there was a need to establish a more suitable solution in the provision of appropriate mental care to the growing number of psycho-traumatized victims of the hostilities.

In 1998, a designated department for emergency mental health services was established in the Ministry of Health. This division was tasked, among other things, with the establishment of a new set of services to treat acute stress and anxiety among civilian casualties of war and terror. As an initial step, professional workers were trained and educated to provide mental first aid. This was achieved through a six-month long course encompassing all aspects of the field. The aim was to achieve a stream-down effect in which course trainees would become trainers themselves and propagate the knowledge to their peers. In a later step, protocols and Standard Operating Procedures (SOPs) were generated to create a national standardized approach to mental health care provision during crisis. To avert the frequent problem of stigmatization associated with the former and obsolete system, a decision was made to treat ASR cases in the Emergency Room (ER) of general hospitals. This was called for because casualties from the terror scene were frequently taken to the ERs, and it was deemed reasonable to have the mental health interventions there as well. To institutionalize this approach, two SOPs were generated. The first dealt with the erection of ER Stress Site (ERSS) during crisis, and the second, with the coupling of specific psychiatric hospitals with specific general hospitals that do not possess a psychiatric ward for assistance with professional caregivers. These procedures outlined certain guidelines for the operation of the ERSS, such as the number and composition of operating staff, the mandatory screening of all patients admitted to the ER for ASR at the ERSS, etc.

The psychotherapy protocol employed in the ERSS concentrated on debriefing of the traumatic event. Victims were encouraged to describe and discuss their experiences as part of the treatment protocol. Since the therapy was taking place within an operating ER, use of medication for tranquilizing distressed patients was common, including the use of Benzodiazepines. It was not until several years later that accumulated experience and scientific research revealed that both methods were counterproductive in preventing ASD and PTSD, and may actually impede recovery [8].

During the second *Intifada* (2000–2003), it became clear that the ERSS system was insufficient in providing a comprehensive solution to the problem. On the one hand, the close proximity of the ERSS to the ER created an unnecessary burden on the medical staff of the ER, and on the other hand, it allowed ERSS admitted patients to be exposed to traumatic images of casualties who were treated in the ER, which usually worsened their condition. As a result, a decision was made to remove the ERSS from the ER to a separate location, still within the hospital premises, in order to mitigate both adverse effects - relieving the workload of the ER staff, and shielding patients admitted to the Stress Site from further exposure to detrimental conditions. These protocols for operating the Stress Site are still valid ordinances in Israeli hospitals for the accommodation of any patient seeking mental relief upon admittance to the ER.

The steep rise in the rate of civilians seeking mental assistance during the Second *Intifada*, coupled with the strict regulations of the national insurance law, which were not in favor of casualties, created a void which numerous Non-Governmental Organizations (NGOs) stepped in to fill. These NGOs provided victims with financial support and relief, and most importantly, offered them mental relief and treatment. To support these activities financially, efforts were made to bring forward the issue of psycho-trauma to the attention of the fundraisers. These efforts led to the establishment of the Israeli Trauma Coalition (ITC), which is comprised of seven leading NGOs in the fields of psycho-trauma. The ITC goals were to provide direct mental aid to patients, to educate and train intervention teams, and to promote resilience among victimized populations across the country [3].

The next major milestone in the evolution of the Israeli mental health care system was registered during the Second Lebanon War (2006). The war demonstrated the extent to which the civilian population in the home front can be victimized in a short period of time. Moreover, the fact that the war affected mostly the northern population of Israel, an area known to be relatively rural, combined with the high-trajectory nature of the threat (e.g. rockets), created a logistical challenge of providing mental care for the population. The Stress Site model at the hospitals was largely inadequate. To overcome this insufficiency, a collaboration between the Ministry of Health and the Israeli Civil Defense Authority (The Home Front Command (HFC) of the Israeli Defense Forces) led to the conceptualization of the Community Stress & Anxiety Center (CSAC) model. The rationale behind this concept was four-fold: (A) the provision of mental care closer to patients’ homes in a communal environment that promoted continuity of care post-crisis; (B) reduction of workload in hospitals in both Stress Sites and ERs; (C) reducing the occupancy duration of ambulances by shortcutting transit distances, thus allowing for the restriction of rocket threats to moving ambulances, while at the same time ensuring the availability of the ambulances for other calls; and (D) reducing the stigmatization associated with being treated for mental distress [9,10].

Two weeks into the armed conflict, the mental health division of the HFC opened five CSACs in the northern region (namely in Carmiel, Ma’alot, Tiberius, Kiryat-Ata and Kibbutz Lohamei HaGeta’ot). The professional personnel in these centers was comprised of mental health and medical officers of the HFC. Later on, rocket threat led to the shut down of local mental health clinics, which diverted help seekers to the CSACs and created a need for additional caregivers to operate these centers. The Israeli Emergency Medical Services (EMS), or Magen-David Adom (Israel’s Red Cross), were guided in the transportation of ASR and anxiety victims to the CSACs instead of hospitals. In a relatively short period, news about these centers spread sufficiently enough so that people sought help in them by themselves. In total, 534 casualties were treated in these centers in the course of a month [9,10].

In spite of the difficulties encountered in pitching the concept of the CSAC to local mayors with the objective of engaging them in logistically supporting these centers, the overall post-war impression of the CSAC model was as a success. Not only that, but another dramatic achievement was accomplished. In the aftermath of the war, the Israeli National Insurance Institute (Israel’s social security) expressed its consent to subsidize a series of a dozen psychological therapy sessions to any casualty without the need to provide evidence of eligibility under the “victim of hostilities” criterion. Usually, without this formal recognition by the state, patients would have difficulties securing financial and other state-subsidized aid and would have to rely on NGOs to provide them with these services. Up until this new regulation by the Israeli National Insurance Institute, it was up to the ITC to financially assist psycho-traumatic victims, which they did with their fundraising of more than 2 million US dollars [3]. Under the dozen-treatment program, services were provided for patients in designated centers established in general hospitals after the war. These centers continued to provide hundreds of people with services for up to three years after the war had ended. Yet despite their success, due to budgetary restrictions, these centers were closed and reopened only following the Gaza conflict of 2012 (see following).

Another significant advancement brought about in the wake of the Second Lebanon War was in the psychotherapy methods employed in the CSACs. Instead of debriefing and pharmaceutically quieting help seekers, a more effective approach of emotional regulation, mental balancing and reinstating normative functioning was adopted. In this approach, much emphasis is placed on patient empowerment and the establishment of an appropriate support structure for each patient. The treatment of a patient begins with a general assessment of his/her condition according to protocols developed by Dr. Ilan Kutz [11]. This assessment allows for the prediction of ASD development down the road, as well as to identify patients in need of closer monitoring. Following patient assessment, a wide array of psychotherapy methods can be employed, including Cognitive-Behavioral Therapy (CBT), Somatic Experiencing (SE) and Eye-Movement Desensitization and Reprocessing (EMDR). Pharmaceutical interventions are sweepingly avoided, except for extreme situations, and even then, Benzodiazepines are not prescribed. In addition, more often than not, the debriefing of the traumatic event would not take place during this first stage of mental care, rather it would be postponed to a later stage of recovery. Lastly, the revised psychotherapy protocols dictate a dyadic approach for childcare, in which large emphasis is placed on parents’ instruction and empowerment to cope with the child in distress. This is done in light of research findings suggesting that better pyscho-indication and self-efficacy perceptions of parents allow for a better recuperation of the distressed child [12].

The good outcome experienced with the CSAC model during the Second Lebanon War promoted the institutionalization of the CSAC concept with a new procedure written and published by the Health Ministry. This procedure laid down the principles of responsibility and authority in establishing and maintaining a CSAC in any given location. The procedure detailed the different approvals needed to be obtained in order to authorize the use of a designated site as a CSAC. For instance, it had to be accessible to ambulances and had to be approved for safety by the HFC. The procedure also described the necessary equipment and staff to operate a CSAC. The original decision was to allocate professional work force from within the health establishment, i.e. hospitals and clinics. Additional assistance by non-medical caregivers was to be allocated by the local welfare and education services. In addition, a certain amount of responsibility was referred to the regional health directors in order to decentralize the system and generate a more efficient management mechanism.

The conclusions drawn in light of the Second Lebanon War also resulted in a new concept for Resilience Centers. The goal of the Resilience Centers was to provide a public resilience build up throughout all three stages of crisis management: (A) prevention - training and educating professionals and volunteers, early identification of vulnerable populations, promotion of mental resilience, etc. (B) response - providing mental health care during emergency in the CSAC model operated by the Resilience Center staff; and (C) recovery - providing ongoing treatment for stress patients in a post-trauma setting similar in concept to that applied in the designated centers that operated in hospitals following the Second Lebanon War.

The original intent was to have a national deployment of these centers, but fiscal constraints allowed for only a few. It was therefore decided to concentrate these efforts in the Gaza Envelope settlements surrounding the Gaza Strip. A tender was published for the proposition of conceptual frameworks. The awarded concept was that of the Ministry of Health and the Israeli Trauma Coalition. The Health Ministry was assigned by the Prime Minister to lead the establishment efforts and to act as the regulator for these proceedings in a joint venture with other governmental ministries. At the end of the process, five Resilience Centers were founded in the Gaza Envelope, namely in in the Regional Council (RC) of Eshkol, RC of Sdot-Negev, RC of Sha’ar Ha’Negev, RC of Hof Ashkelon and the city of Sderot. In other regions of the country, the task of everyday provision of mental health treatment was assigned to independent NGOs that were operating under the umbrella of the ITC [3].

By the end of 2008, the concept of the Resilience Centers was put to test with the eruption of the first in a series of three Gaza conflicts to date. During this operation, nicknamed “Cast Lead”, the Resilience Centers shifted from prevention to response mode and provided mental care to patients seeking their service. To overcome the surge in demands, an additional nine CSACs were opened in cities and towns in the Gaza Envelope region. Regional psychiatric hospitals assisted with professional caregivers, and the four Health Management Organizations (a.k.a. “sick funds”) supplemented general practitioners who performed physical examination of patients. Overall, these 14 centers and sites operated effectively and maintained their operations in accordance with procedures and treatment protocols. As a matter of fact, these centers were so effective that a noticeable change in the hospital-to-CSAC distribution of patients was generated. During the Second Lebanon War, only a third of the patients attended the CSACs (as opposed to the two-third that attended hospitals), whereas during “Cast Lead” operation, these ratios were swapped and two-thirds of patients attended the CSACs.

However, the “Cast Lead” operation did not go by without difficulties and challenges. First, the intervention teams were faced with a substantial challenge in the accommodation of their treatment protocols designed for a single session to returning patients, i.e., patients who were treated in the past and were triggered to seek mental care due to revisited trauma. Second, the psychiatric hospitals assisting with professional work force complained of shortages in labor for their routine work. Third, CSACs staff were forced to travel long distances in non-protected vehicles, making their experience stressful on its own. Fourth, on numerous occasions, CSACs staff pointed out that occasionally, there was a significant drop in casualty admittance to the CSAC, causing temporary unemployment and a waste of labor. Lastly, Resilience Centers positioned in low density regional councils reported difficulties of the population to reach and return from these centers, especially under rocket threats. To overcome this problem, a decision was made during the course of the conflict to generate localized capabilities to provide initial care in each township, mainly through local social services, and to use the Resilience Center as a back post from which teams could be dispatched upon request.

In the aftermath of “Cast Lead” operation, a systematic process was initiated to map and designate appropriate sites around the country to serve as CSACs. To date, 56 sites have been selected, examined and approved. Efforts are made to recruit and integrate professional teams of health and welfare workers, and to have them trained and ready to operate these centers in the occurrence of future crisis. In addition, following the “Cast Lead” operation, the Ministry of Health initiated a campaign to appoint sites that could serve as designated centers for ongoing treatment, in a similar manner to the concept employed during the Second Lebanon War. Once a patient finalizes the treatment at the CSAC, he is handed a form filled and signed by the caregiver. This form indicates what type of intervention the patient underwent, whether or not the patient requires further treatment, and the caregiver’s recommendations for this ongoing treatment. Citizens can approach the designated treatment centers, which are available across the country, with this form and obtain the required therapy. The appointment of these treatment centers is a joint venture of the ministry with the Israeli National Insurance Institute and is conducted in a manner that ensures that patients would be eligible for the subsidized dozen treatments. This is an ongoing effort to reinstate the former successful program that was awarded to psycho-trauma victims following the Second Lebanon War.

The second time the Emergency Mental Health Services array was put to the test was in 2012 when the “Pillar of Defense” operation was initiated in the Gaza Strip. The response during this crisis was similar to that observed in the previous conflict. However, with the introduction of the “Iron Dome” missile defense system into the battlefield, an overall reduction in help-seeking rates was observed. On the other hand, telephone-based mental assistance (e.g. “hotlines”) rose. People seemed to prefer having their tensions relieved in the comfort and assurance of their own homes instead of having to travel to a nearby, designated center. This phenomenon necessitated that call centers pay closer attention to the mental distress situation of callers and refer those in a more serious condition to the CSACs (or alternatively dispatch the CSAC staff to the caller) in order to allow for adequate psychological intervention.

In addition, operation “Pillar of Defense” was a turning point with respect to the handling of work force. Similarly to “Cast Lead,” intervention teams continued to complain of unemployment during specific hours of the day, especially during the night. Additionally, complaints were made concerning the unnecessary mandatory presence of a psychiatrist in each shift. Consequently, shifts were reduced from a 24/7 to a 08:00–20:00 format, and a surge capacity approach was adopted in which staff numbers were reduced and could be increased upon demand. The presence of a psychiatrist in each CSAC working shift was made optional.

With the dust settling after the “Pillar of Defense” operation, a rethink of operations was done concerning the appointment of responsibility over the management of the emergency mental health system. In a series of deliberations, the decision was made to shift the responsibility from the Ministry of Health to the local authorities, leaving the first to act solely as a regulator. The CSAC model was refurbished and was given a new name - Mental Health Support Centers (MHSC) [13]. Acknowledging the fact that local mayors and heads of regional councils were more capable in understanding their local public and managing its resources, they were assigned with the responsibility to overlook the process of the establishment of MHSCs in their respective municipalities. Nevertheless, this shift in responsibility was greeted with ambivalence, largely because governors assumed different levels of responsibility, resulting for some in an ever more political decision-making process.

The new model was put to the test during the recent “Protective Edge” operation (July-August, 2014). While some claimed some “chaos” in the system, noticeable mostly through the regression in hospital-to-MHSC distribution rates achieved in the previous conflict, some argued that overall, this new approach was justified. Those favoring this notion referred to the continued overall drop in attendance rates to stress-relief sites and the introduction of tele-media therapy in two centers in the city of Netivot and the RC of Eshkol. The latter allowed for the provision of mental care to remote settlements next to the Gaza border that had been otherwise inaccessible.

In order to balance the system, the department of Emergency Mental Health Services in the Ministry of Health was tasked with a regulatory role to oversee the system as a whole, and was also responsible for the provision of training and educational programs to maintain eligibility among caregivers. Each site established for the provision of mental health care underwent such training, and at each site, a point-of-contact was designated to be responsible for maintaining this capability.

### The current status and future challenges

The current model of the Emergency Mental Health Services in Israel can be divided into three components: (a) immediate, on-site intervention administered by local teams; (b) Mental Health Support Centers (MHSCs) operating independently or as part of a Resilience Center to provide readily available, accessible, stigma-free treatment to anyone experiencing mental distress or anxiety; (c) on-going efforts to promote public resilience during routine times through Resilience Centers and other NGOs.

The emergency mental health system, which has been established in Israel over the course of forty years, has grown through a systematic process of lesson learning from actual experiences with threats. It would be fair to argue that this process of maturation has placed the Israeli system in an example-setting position to other countries embarking on a quest of generating a similar mechanism. It is important to note that the emergency mental health provision in Israel has rooted itself in solid grounds by means of change in its discourse. Terms such as anxiety and stress are replaced with terms such as mental support. This is not merely semantics. This change constitutes a profound understanding of the complexity of mental health provision to victimized populations over a prolonged period, and encompasses all the different aspects of mental distress and hardship presented by different people during crisis. It also covers the recent developments in treatment administration through ever advancing means, such as telecommunication, and making mental aid an accessible commodity to the public [14]. It is the intention of the Ministry of Health to develop the tele-media capabilities demonstrated in the last Gaza conflict, which proved highly effective in providing mental health care to victims.

Nonetheless, the Israeli emergency mental health system faces several challenges in the near future. As described earlier, in the aftermath of the last conflict in Gaza, it became clear that alterations in the current structure of the system are necessary. These changes will mostly draw from previous architectures of the system in order to retrieve elements that were beneficial in the past. In particular, there is a need for an efficient process of integration between the local authorities and the different governmental agencies in order to ensure continuity, and promote the cooperation around the MHSC concept.

Lastly, efforts should be invested in establishing a national training organization responsible for preparing and training local MHSC teams. This step would help in generating a comprehensive approach to the harmonization of the mental health system in Israel to the benefit of its users.

## Appendix A

**Brief description of the armed conflicts presented in the paper from the Israeli perspective**^**a**^

**1979 Naharya terror attack**

The 1979 Nahariya attack (codenamed by its perpetrators as the Nasser Operation) was a raid by four *Palestine Liberation Front* (PLF) militants in Nahariya, Israel on April 22, 1979. The group, led by 16-year-old Samir Kuntar, used a small, 55 horsepower (41 kW) boat to travel from Tyre, Lebanon to Israel. The raid resulted in the deaths of four Israelis, including a father and two of his young children. This terror attack is regarded as one of the most brutal terror attacks in the history of Israel.

**1980’s northern border attacks**

On 6 June 1982, Israel invaded Lebanon for the purpose of attacking the *Palestine Liberation Organization* (PLO), giving rise to the 1982 Lebanon War. From January 1984 until 1997, numerous launchings of rockets and mortars and nine incidents of terrorist invasions into Israeli northern settlements were registered. The worst incident took place on 25 November 1987 and was nicknamed *“Night of the Gliders”* in which two Palestinian guerrillas infiltrated into Israel from South Lebanon using hang gliders to launch a surprise attack against the Israel Defense Forces (IDF). Earlier conflicts prior to the 1982 Israeli invasion, include *Operation Litani*’s attempt to eradicate the PLO bases from Lebanon and support Christian Maronite militias, following constant attacks from the PLO on the civilian population of Galilee (Northern Israel). The 1982 invasion resulted in the creation of the Security Zone and the PLO departure from Lebanon. The creation of the Security Zone in South Lebanon has benefited the civilian Israeli population as the Galilee has suffered less violent attacks by *Hezbollah* (9 Israeli civilians killed and at least 248 wounded) than previously by PLO in the 1970s (hundreds of Israeli civilian casualties). Despite Israel’s success in eradicating PLO bases and partial withdraw in 1985, the Israeli invasion had actually increased the severity of conflict with local Lebanese militias and resulted in the consolidation of several local Shia Muslim movements in Lebanon, including *Hezbollah* and *Amal*, from a previously unorganized guerrilla movement in the south. Over the years, military casualties of both sides grew higher, as both parties used more modern weaponry, and *Hezbollah* progressed in its tactics. By the early 1990s, *Hezbollah*, with support from Syria and Iran, emerged as the leading group and military power, monopolizing the directorship of the guerrilla activity in South Lebanon.

**The first Intifada (1987–1991)**

The First Intifada was a Palestinian uprising against the Israeli occupation of the Palestinian Territories, which lasted from December 1987 until the Madrid Conference in 1991, though some date its conclusion to 1993, with the signing of the Oslo Accords. The uprising included general strikes, boycotts of Israeli civil administration institutions in the Gaza Strip and the West Bank, civil disobedience in the face of army orders, and an economic boycott consisting of refusal to work in Israeli settlements on Israeli products, refusal to pay taxes, refusal to drive Palestinian cars with Israeli licenses, graffiti, barricading, and widespread throwing of stones and Molotov cocktails at the IDF and its infrastructure within the Palestinian territories. Over six years the Israeli Defense Forces killed an estimated 1,162–1,204 Palestinians while Palestinians killed 100 Israeli civilians and 60 IDF personnel and injured more than 1,400 Israeli civilians and 1,700 soldiers.

**The First Gulf War (1991)**

The Gulf War (2 August 1990 – 28 February 1991), was a war waged by coalition forces from 34 nations led by the United States against Iraq in response to Iraq’s invasion and annexation of Kuwait. As part of the armed conflict, Iraq launched Scud missiles against Coalition military targets in Saudi Arabia and against Israel in hope to provoke a military response from Israel. The Scud missiles targeting Israel were relatively ineffective, as firing at an extreme range resulted in a dramatic reduction in accuracy and payload. According to the Jewish Virtual Library, a total of 74 Israelis died as a result of the Iraqi attacks: two directly and the rest from suffocation and heart attacks. Approximately 230 Israelis were injured. Extensive property damage was also caused, and according to Israel Ministry of Foreign Affairs, “Damage to general property consisted of 1,302 houses, 6,142 apartments, 23 public buildings, 200 shops and 50 cars.” It was feared that Iraq would fire missiles filled with nerve agents such as sarin. As a result, Israel’s government issued gas masks to its citizens. When the first Iraqi missiles hit Israel, some people injected themselves with an antidote for nerve gas. It has been suggested that the sturdy construction techniques used in Israeli cities, coupled with the fact that Scuds were only launched at night, played an important role in limiting the number of casualties from Scud attacks.

**The second Intifada (2000–2003)**

The Second Intifada, also known as the *Al-Aqsa Intifada*, was the second Palestinian uprising against Israel – a period of intensified Israeli-Palestinian violence. It started in September 2000, when Ariel Sharon (later the prime minister of Israel) made a visit to the Temple Mount, seen by Palestinians as highly provocative. Both parties inflicted high numbers of casualties among civilians as well as combatants: the Palestinians by numerous suicide bombing and gunfire; the Israelis by tank and gunfire and air attacks, by numerous targeted killings, and by harsh reactions on demonstrations. The death toll, including both military and civilian, is estimated to be about 3,000 Palestinians and 1,000 Israelis, as well as 64 foreigners. Some consider the *Sharm el-Sheikh* Summit on February 8, 2005, the end of the Second Intifada, when President Mahmoud Abbas and Prime Minister Ariel Sharon agreed to stop all acts of violence against Israelis and Palestinians and reaffirmed their commitment to the Roadmap for peace.

**The Second Lebanon War (2006)**

The 2006 Lebanon War, also called the Second Lebanon War, was a 34-day military conflict in Lebanon, northern Israel and the Golan Heights. The principal parties were *Hezbollah* paramilitary forces and the Israeli military. The conflict started on 12 July 2006 and continued until a United Nations-brokered ceasefire went into effect in the morning of 14 August 2006, though it formally ended on 8 September 2006 when Israel lifted its naval blockade of Lebanon. The conflict was precipitated by the *Zar’it-Shtula* incident on 12 July 2006, resulting with the abduction of two Israeli soldiers by Hezbollah to Lebanon. *Hezbollah* demanded the release of Lebanese prisoners held by Israel in exchange for the release of the abducted soldiers. Israel refused and responded with airstrikes and artillery fire on targets in Lebanon followed by a ground invasion of southern Lebanon. During the war, *Hezbollah* fired between 3,970 and 4,228 rockets at a rate of more than 100 per day, unprecedented since the Iran-Iraq war. An estimated 23% of these rockets hit cities and built-up areas across northern Israel, while the remainder hit open areas. One million Israelis had to stay near or in bomb shelters or security rooms, with some 250,000 civilians evacuating the north and relocating to other areas of the country. The conflict is believed to have killed between 1,191 and 1,300 Lebanese people and 165 Israelis.

**Operation “Cast Lead” (2009)**

The Gaza War, also known as Operation Cast Lead, was a three-week armed conflict between Palestinians in the Gaza Strip and Israel that began on 27 December, 2008 and ended on 18 January, 2009 in a unilateral ceasefire. Israel’s stated goal was to stop rocket fire into Israel and weapons smuggling into the Gaza strip. The strike range of Hamas rockets had increased from 16 km (9.9 mi) to 40 km (25 mi) since early 2008 with the use of improved Qassam and factory-made rockets. As of January 13, 2009, Palestinian militants had launched approximately 565 rockets and 200 mortars at Israel since the beginning of the conflict, according to Israeli security sources. The rockets killed three civilians and one IDF soldier and wounded 182 people, with another 584 people suffering from shock and anxiety. Besides being hit with rockets fired from Gaza, Israel experienced other attacks along the borders with Lebanon and Syria.

**Operation “Pillar of Defense” (2012)**

Operation Pillar of Defense was an eight-day Israel Defense Forces (IDF) operation in the *Hamas*-governed Gaza Strip, which began on 14 November 2012 with the killing of Ahmed Jabari, chief of the Gaza military wing of *Hamas*. The operation was preceded by a period with a number of mutual Israeli–Palestinian responsive attacks. During the operation, *Hamas*, the *Al-Qassam Brigades* and the *Palestinian Islamic Jihad* (PIJ) further intensified their rocket, firing over 1,456 rockets into Israel. About 421 rockets were intercepted by Israel’s Iron Dome missile defense system, another 142 fell on Gaza itself, 875 fell in open areas, and 58 hit urban areas in Israel. By the end of the operation, six Israelis had been killed, two hundred forty were injured. In addition, a bus in Tel-Aviv was bombed by an Arab-Israeli, injuring 28 civilians.

**Operation “Protective Edge” (2014)**

Operation Protective Edge was a seven-week conflict in the *Hamas*-governed Gaza Strip, which began on 8 July 2014. The stated aim of the Israeli operation was to stop rocket fire from Gaza into Israel, which increased after an Israeli crackdown on Hamas in the West Bank was launched following the kidnapping and murder of three Israeli teenagers by two Hamas members. As in previous conflicts, the Palestinian terror groups launched rockets and mortars into Israel, reaching record breaking ranges with rockets fired all the way to Haifa in the north. According to the IDF, of all the 4,564 projectiles fired at Israel, 224 hit built-up areas, 735 were intercepted by the Iron Dome, 875 fell inside Gaza and the rest fell in open territory or failed to launch. In Israel, an estimated 5,000 to 8,000 citizens temporarily fled their homes due to the threat of rocket and mortar attacks. Between 2,127 and 2,192 Gazans were killed (including 513 children) and between 10,895 and 11,100 were wounded. Sixty-six Israeli soldiers, 5 Israeli civilians (including one child) and one Thai civilian were killed, as well as 469 IDF soldiers and 261 Israeli civilians that were injured.

## Appendix B

**Additional Roles of the Mental Health Services during Emergencies**

The department of emergency mental health services in the Ministry of Health is tasked with additional responsibilities. First, it is involved in international relief aid during crises by supporting the mental health aspects of Israeli relief delegation. Examples for such contributions are the 2004 Sri Lanka Tsunami disaster, The 2004 Beslan (Russia) school hostage crisis, and the 2005 New-Orleans disaster following Hurricane Katrina.

Second, the department assist the ministerial decision making process concerning mass-psychology in crisis situation. The most recent example for this is the ongoing Ebola crisis (2014), which necessitated risk communication adaptation to prevent and mitigate potential psycho-trauma effects to the public. This was also the case with the SARS pandemic in 2003, the Avian Flu scare in 2005 and the Swine Flu pandemic in 2009.

Lastly, the division oversees the ongoing recovery efforts of pyscho-trauma victims post-crisis. This activity involves doctrinal development and procedural work, designating and training professional workforce, relief work in assisting the recuperation of patients towards overcoming their trauma, e.g., by initiating tension-abatement vacations outside of threat zones, and “help the helpers” programs to assist stress remission among mental health providers and promote their resilience in future experiences with this complicated, but important, work.

## Endnotes

^a^Information retrieved from Wikipedia. http://en.wikipedia.org/. Accessed January 11th, 2015.

## Abbreviations

ASD: Acute Stress Disorder
ASR: Acute Stress Reaction
CSAC: Community Stress & Anxiety Center
EMS: Emergency Medical Services
ER: Emergency Room
ERSS: Emergency Room Stress Site
HFC: Home Front Command
ITC: Israeli Trauma Coalition
MHSC: Mental Health Support Centers
NGO: Non-Governmental Organization
PTSD: Post-Traumatic Stress Disorder
RC: Regional Council
SOP: Standard Operating Procedure
